# Examples of sequence conservation analyses capture a subset of mouse long non-coding RNAs sharing homology with fish conserved genomic elements

**DOI:** 10.1186/1471-2105-14-S7-S14

**Published:** 2013-04-22

**Authors:** Swaraj Basu, Ferenc Müller, Remo Sanges

**Affiliations:** 1 Laboratory of Animal Physiology and Evolution, Stazione Zoologica Anton Dohrn, Villa Comunale, 80121, Naples, Italy; 2 Centre for Rare Diseases and Personalized Medicine, School of Clinical and Experimental Medicine, College of Medical and Dental Sciences, University of Birmingham, Birmingham, UK

## Abstract

**Background:**

Long non-coding RNAs (lncRNA) are a major class of non-coding RNAs. They are involved in diverse intra-cellular mechanisms like molecular scaffolding, splicing and DNA methylation. Through these mechanisms they are reported to play a role in cellular differentiation and development. They show an enriched expression in the brain where they are implicated in maintaining cellular identity, homeostasis, stress responses and plasticity. Low sequence conservation and lack of functional annotations make it difficult to identify homologs of mammalian lncRNAs in other vertebrates. A computational evaluation of the lncRNAs through systematic conservation analyses of both sequences as well as their genomic architecture is required.

**Results:**

Our results show that a subset of mouse candidate lncRNAs could be distinguished from random sequences based on their alignment with zebrafish phastCons elements. Using ROC analyses we were able to define a measure to select significantly conserved lncRNAs. Indeed, starting from ~2,800 mouse lncRNAs we could predict that between 4 and 11% present conserved sequence fragments in fish genomes. Gene ontology (GO) enrichment analyses of protein coding genes, proximal to the region of conservation, in both organisms highlighted similar GO classes like regulation of transcription and central nervous system development. The proximal coding genes in both the species show enrichment of their expression in brain. In summary, we show that interesting genomic regions in zebrafish could be marked based on their sequence homology to a mouse lncRNA, overlap with ESTs and proximity to genes involved in nervous system development.

**Conclusions:**

Conservation at the sequence level can identify a subset of putative lncRNA orthologs. The similar protein-coding neighborhood and transcriptional information about the conserved candidates provide support to the hypothesis that they share functional homology. The pipeline herein presented represents a proof of principle showing that a portion between 4 and 11% of lncRNAs retains region of conservation between mammals and fishes. We believe this study will result useful as a reference to analyze the conservation of lncRNAs in newly sequenced genomes and transcriptomes.

## Background

Long non-coding RNAs (lncRNAs) were firstly reported as transcripts expressed in large numbers in mammalian transcriptomes [1,2]. They were shown to constitute more than half of all the transcriptional outputs of mammalian genomes [3]. Prior to these reports *Xist *in mammals and *Xlsirt *in amphibians were the only well characterized lncRNAs described to function in Χ; chromosome inactivation and the formation of cytoskeleton [4,5]. Recently, several aspects of development and disease have been associated with lncRNAs function albeit to a small proportion of them. Processes associated with lncRNAs include epigenetic regulation of multi-gene loci [6,7], apoptosis and cell cycle [8], regulation of gene splicing [9] and tumor suppressor activity [10]. There are also examples of lncRNAs playing a role in the adaptive immunity of mammals [11], being differentially expressed in response to carcinogens [12] and functioning as enhancers [13].

A probe into the functional role of lncRNAs requires knowledge of the specificity of their expression across developmental stages and different tissues. Microarray technology was initially used to detect lncRNAs in differentiating mouse embryonic stem cells. Many lncRNAs showed correlation of expression with the flanking protein-coding genes that associated with developmental functions [6]. Similar transcriptomics studies identified many lncRNAs expressed in CD8+ cells and during neuronal lineage specification in mouse [11,14]. However, cross-hybridization, background noise and limited genome coverage associated to array based detection techniques were often used as arguments against the pervasive existence of lncRNAs. Today these problems have been solved thanks to the advent of the RNA sequencing technology (RNAseq) [15]. RNAseq deals with direct quantification of a cDNA population and is not limited to transcripts mapped to known genomic sequences. An RNAseq experiment on differentiating human neurons led to the identification of about 1,600 lncRNAs with dynamical expression levels [16]. Recently, sequencing studies have even identified hundreds of lncRNAs expressed during early developmental stages in zebrafish and *C. elegans *finally demonstrating that the pervasiveness of non-coding transcription is not an exclusive feature of mammalian transcriptomes. Pauli *et al *identified about 1,100 non-coding RNAs expressed during embryogenesis in zebrafish [17]. Ulitsky *et al *reported a set of about 700 long intergenic non-coding RNAs (lincRNAs) expressed during zebrafish development; they show low sequence similarity but conserved genomic locations with their mammalian counterparts [18]. Finally Nam *et al *reported a catalog of 230 lncRNAs in *C.elegans *[19] which show similar features to vertebrate lncRNAs in terms of lack of general sequence conservation as well as stage specific expression patterns. The execution of large-scale studies about the discovery and characterization of candidate lncRNAs is facilitating the establishment of a catalog of these important molecular players which, in turn, permit to annotate them with specific biological validations and comparative analyses improving our knowledge of the lncRNAome [20].

However, lack of sequence homology and lack of deep tissue and stage specific expression data have been one of the obstacles in defining a proper catalog of lncRNAs among different species. Computational methods for the discovery and the annotation of lncRNAs are sparse and mainly limited to mammalian genomes. Parameters like open reading frame length, lack of homology to protein-coding genes and protein domains, nucleotide composition and substitution rates have been used previously to define computational lncRNA discovery pipelines [21-24]. Sequence conservation has not been often used as support to justify the presence of lncRNAs in an organism because the lncRNAs identified in human, mouse, zebrafish and *C. elegans *showed little or no sequence conservation in the majority of the population [17-19,25]. In addition, Pauli *et al *stated that the level of conservation for the majority of zebrafish lncRNA is comparable to that of introns and that only few of them are really conserved [17]. However, short spans of sequence conservation for specific lncRNAs were reported [18]. Four evolutionary constrained mouse lncRNAs were shown to be conserved in sequence and expression between mouse, opossum and chicken [26]. Forty three putative long non-coding sequences from chicken ESTs were found to share sequence homology with human, rat and mouse transcripts [27]. *Xist*, the lncRNA responsible for the Χ chromosome inactivation in eutherian mammals shares sequence homology in 14 vertebrates [28]. *Sox2ot *(*Sox2 *overlapping transcript) and *Har1F *(Human accelerated region *1F*) are conserved amongst vertebrates [29,30]. The biggest group of lncRNAs constrained in terms of their nucleotide substitution rates between mouse and human is represented by a set of 659 mouse transcripts [31]. This set showed a tendency to juxtapose genes involved in the regulation of transcription and development. A subset of these lncRNAs expressed in the mouse brain and defined as *CNS-specific *appear to show tissue specific expression pattern similar to their proximal protein coding genes. Despite these evidences, a lack of general sequence conservation associated with lncRNAs has been proposed and this becomes more evident when considering long evolutionary distances such as the one separating mammals from fishes, even if fragments of sequence conservation have been detected [18,27].

Despite these evidences, a systematic analysis of sequence conservation of vertebrate lncRNAs is still lacking and it remains unclear whether and to what extent there is general sequence conservation of lncRNAs between mammals and fishes. In order to fulfill this lack, the work here presented tries to define the level of conservation of mouse lncRNAs in the zebrafish genome using an unbiased choice for the comparison parameters, taking advantage of randomizations and receiver operating characteristic (ROC) analyses. Our approach is centered around subsets of mouse constrained and/or well annotated lncRNAs, based on the assumption that such transcripts constitute a representative set of lncRNAs ideally containing small amounts of transcriptional noise. We would like to specify that, although our analysis consider a subset of already published and very well annotated lncRNAs, we cannot rule out the possibility that some of them might actually be coding for short peptides. Recent studies suggest that few candidate lncRNAs can produce short peptides and there may exist a class of bifunctional RNAs encoding both mRNAs and functional noncoding transcripts [32-34]. Therefore, specific biological validations remain a fundamental step for a proper characterization of these elements. Nevertheless, the special care taken in the choice of the datasets to analyze and the literature about them, makes us confident that we are currently using, the best sets of candidate lncRNAs. We compared these transcripts against the set of zebrafish phastCons elements [35] reported to be significantly conserved among fishes. The phastCons program uses a hidden Markov model-based method that estimates the probability that each nucleotide belongs to a conserved element, based on multiple alignments of selected species. We used the phastCons6way track to select elements conserved among fishes. These are based on scores built on multiple alignment of the zebrafish genome with tetraodon, stickleback, human, mouse and western clawed frog. It is important to point out here that, considering the way in which they are built, these elements represent the best selection of sequences conserved, in first instance, among fishes, but many of them can also result conserved among vertebrates. This choice implicitly adds more genomes to our analyses and is based on the assumption that lncRNAs conserved between mouse and zebrafish are expected to be primarily conserved among teleosts. For this pilot study, the reduction in the dataset dimension, given by such choice, limited the zebrafish genomic search space to the phastCons sequences, rather than to the full genome, making it feasible to use several randomizations steps (shuffling of the query sequences) to specifically identify the levels of conservation proper of lncRNAs.

Here we show that the usage of the BLASTn e-value and alignment length as cut-offs is sufficient to distinguish conservation of mouse lncRNAs against zebrafish phastCons elements as compared to shuffled sequences. From an initial dataset of about 2,800 mouse lncRNAs we demonstrate that between 4 and 11% of them contain fragments significantly conserved in zebrafish in agreement with the results by Ulitsky et al [18] on a smaller dataset. Gene ontology enrichment analyses of protein-coding genes flanking the conserved elements, identified similar functional classes significantly enriched in both species, such as regulation of transcription and development. These coding genes exhibited enrichment for expression in the brain in both mouse as well as zebrafish. The lncRNAs shown to be conserved are deemed to be functionally important and suggested for further experimental validation of their function.

## Results and discussion

### Selection of conservation parameters to select significantly conserved lncRNAs

**Figure 1:**
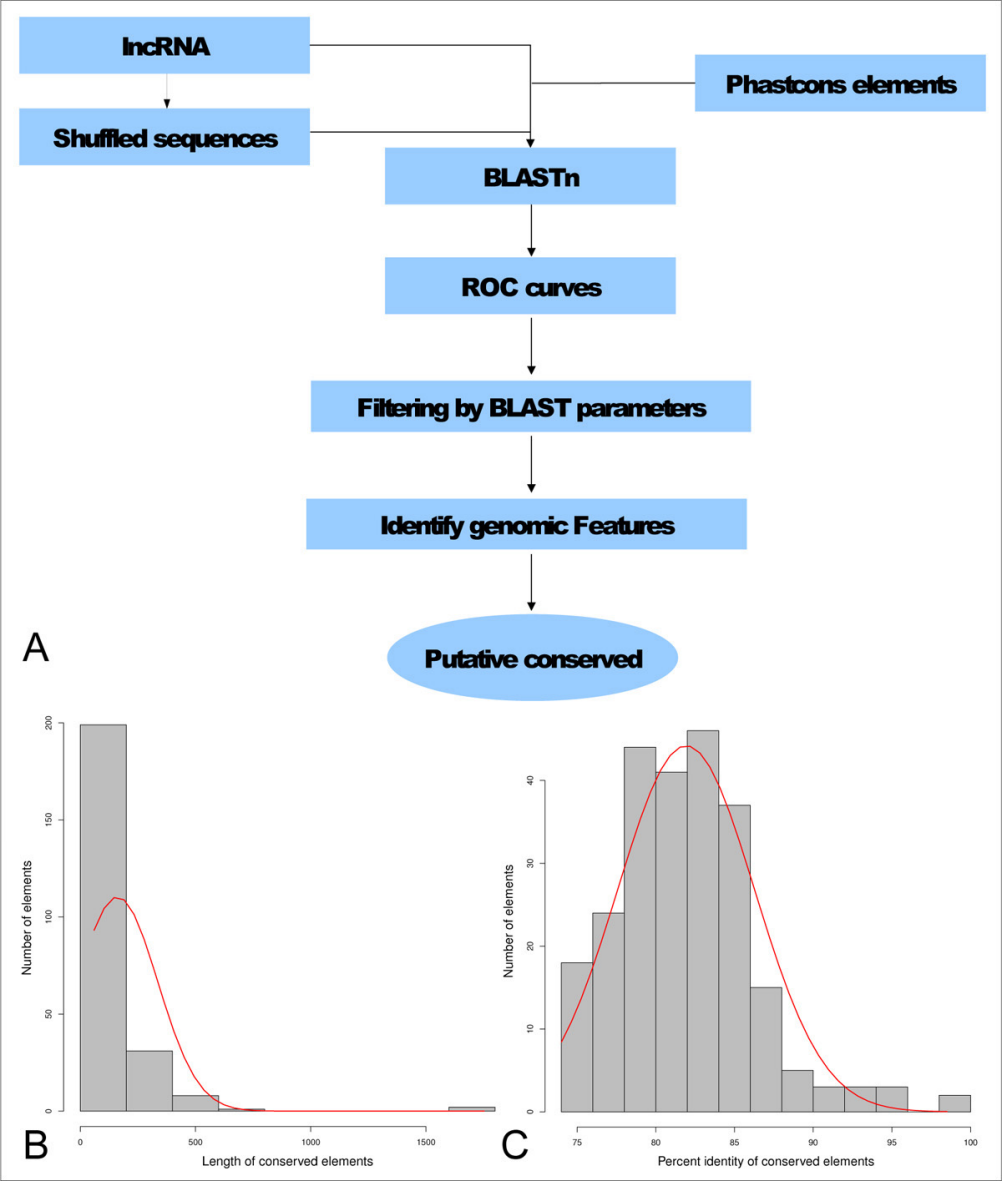
**Pipeline to detect lncRNA sequence conservation and descriptive statistics of the identified conserved elements**. **A) **Schematic representation of the pipeline created to identify putative conserved mouse long non-coding RNAs in the zebrafish phastCons elements. **B) **Distribution of lengths of the identified conserved elements **C) **Distribution of percentage identities of the identified conserved elements.

**Figure 2:**
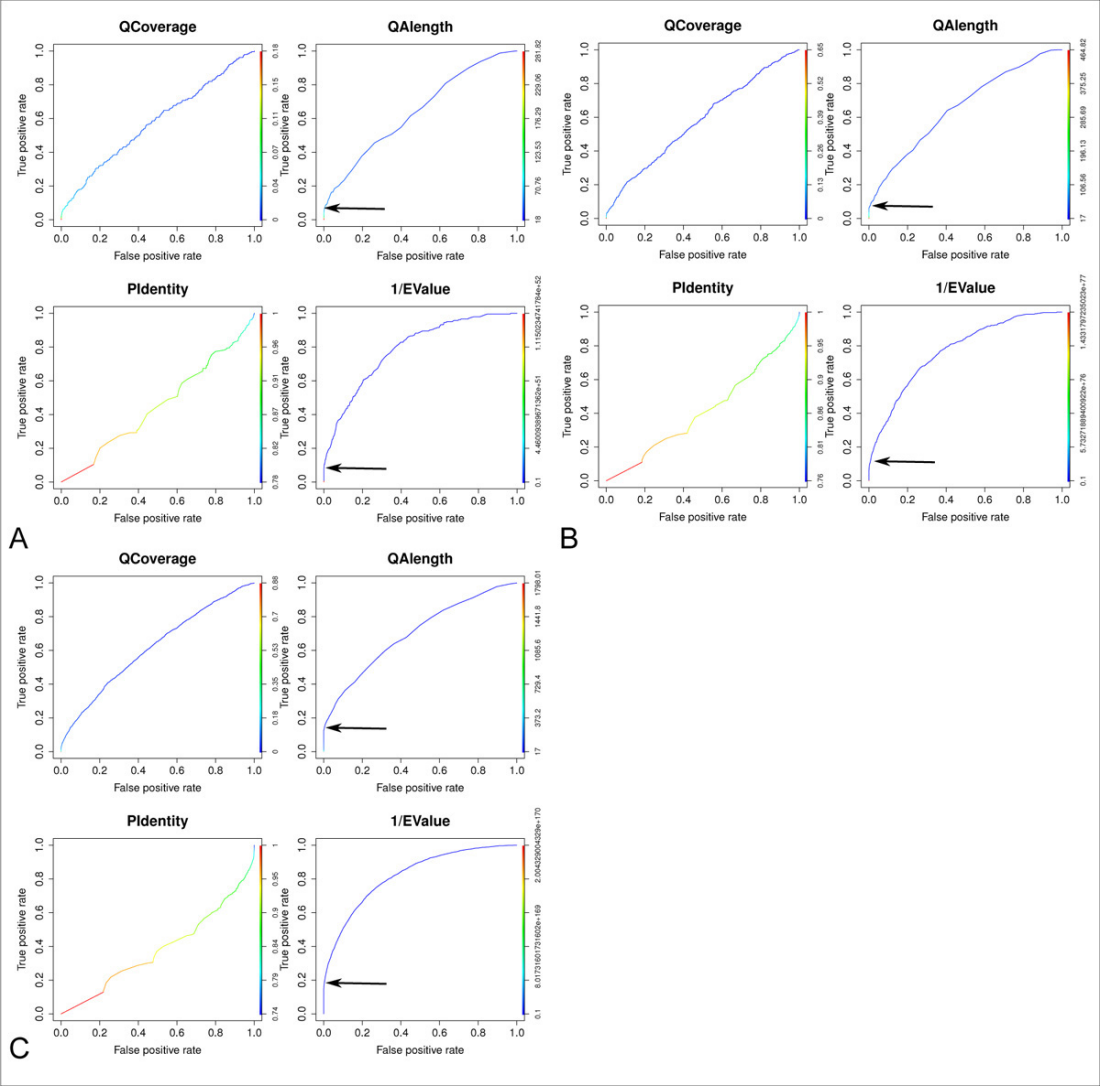
**ROC curves of CNS, NCNS and Ensembl datasets homology search results**. The receiver operating characteristic (ROC) curve plots the true positive rate against the false positive rate for the different possible cut points of specific variables of the BLASTn results. The true positive rate is measured by the BLASTn search of lncRNAs against the phastCons elements while the false positive rate accounts for the BLASTn search of shuffled sequences against the phastCons elements. The ROC curves were used to determine the ideal score for a cut point which may separate the alignments with biological significance from the random occurring alignments. ROC curves for query coverage (QCoverage), percentage identity (PIdentity), query alignment length (QAlength) and e-value (1/EValue) at word size 11 for **A) **CNS dataset **B) **NCNS dataset, **C) **Ensembl dataset. The cut-off for a parameter is defined as the point of steep incline in the true positive rate as compared to the false positive rate. The significant cut-off defined in the present analysis are indicated by arrows. ROC curves for the e-value parameter in the plots show the reciprocal of the e-value (1/e-value) because plotting the e-value produced curves sensibly skewed below the diagonal line.

We developed a pipeline to identify conserved mouse lncRNA fragments in zebrafish using sequence identity, randomization and the identification of an unbiased threshold to detect significant levels of conservation (Figure 1 A). In order to identify the optimal parameters capable to select conserved lncRNA sequences, we used receiver operating characteristic (ROC) like analyses on the distribution of the following BLASTn alignments result measures: 1) query coverage, 2) query alignment length, 3) percentage identity and 4) e-value. ROC like analyses were performed on the results of the following BLASTn searches: 1) mouse lncRNA against zebrafish phastCons elements (true positive set), 2) shuffled mouse lncRNA sequences against zebrafish phastCons elements (false positive set). In order to select significant results we defined a specific cut-off showing less than 0.05% false discovery rate (FDR) for each parameter. The analysis was applied to different datasets and, after the application of the identified filter, between 4 to 11% of the sequences in the true positive datasets resulted to be significantly conserved. Conserved fragments show a mean length of about 160 nucleotides and an average identity of about 80% with their corresponding mouse lncRNA fragments (Figure 1B and 1C). Specifically, mouse candidates lncRNAs from two sources representing three datasets, were used to determine sequence conservation. Mammalian constrained lncRNAs from mouse (659 transcripts defined as CNS/NCNS dataset) [31] were divided into Central Nervous System specific (239 CNS transcripts) and non-CNS specific (420 NCNS transcripts) giving rise to the first and second datasets. LncRNAs identified in the mouse genome by the Ensembl lincRNA annotation pipeline [36,37] (2,147 EnsEMBL transcripts, EnsEMBL version 62) formed the third dataset. An initial exploratory analysis was performed by using BLASTn with word lengths ranging from 8 to 11 nucleotides on the CNS dataset. ROC curves, plotting the distributions of the indicated measures (Figure 2 A) suggest that the reciprocal of the e-value (1/e-value) is the factor capable to better segregate results between the true positive and false positive sets (area under curve, AUC = 0.79). We plot the reciprocal of the e-value (1/e-value) because plotting the e-value produced curves significantly skewed below the diagonal line [38]. In addition, alignment length presents an AUC of 0.64 and, at a manual inspection of results, we noticed that this measure is capable to filter out low complexity (repeated) regions that, in few results, show multiple hits with a small e-value and are hence retained by the exclusive e-value filter. It is now becoming evident that repeats are enriched in lncRNAs [39,40] At the light of such considerations, we decided to combine the 2 measures in order to select significantly conserved lncRNAs, avoiding to obtain low complexity regions in the set of results. Combining the two parameters for filtration gave us zero false positives for each dataset. Interestingly, the change in word size does not affect the performance of the classifier (Additional File 1). Therefore, word size of 11 nucleotides is used in all subsequent analyses. We selected as significantly conserved, lncRNAs sequences showing <0.05% FDR for each of the 2 parameter (e-value and alignment length). Cut-off values were calculated in order to consider significant a percentage of false positives smaller than 0.05% when the same filter is applied to the randomized data. An e-value cutoff of 5e-05 and an alignment length cut-off of 70 nucleotides satisfied this criteria resulting in 11 lncRNAs from the CNS dataset significantly conserved within the zebrafish phastCons elements (Table 1). The BLASTn search was repeated for the NCNS and the Ensembl datasets (Table 1). ROC curves (Figure 2 B, C) and manual inspection of data confirmed the e-value and query alignment length as the best parameters to successfully identify significantly conserved lncRNAs (AUC NCNS: e-value 0.76, alignment length 0.66; AUC EnsEMBL: e-value 0.82, alignment length 0.70). The identified cutoffs are as follows: NCNS) e-value 1e-04, alignment length 66; EnsEMBL) e-value 2e-04, alignment length 62. The results and the annotations of the homology searches for all 3 datasets can be found in Additional File 2. We also performed secondary structure analyses to test if we could segregate the false and true positives more efficiently using RNAz on the BLASTn alignments. RNAz is an efficient method for detecting functional RNAs combining comparative sequence analysis and structure prediction. The program performs two basic calculation: 1) the measure for RNA secondary structure conservation and 2) the measure for thermodynamic stability [41]. The 3 RNAz result parameters used to build the ROC curves were: ratio of pairwise identity by sequence conservation index, Z score and P value (1/P value) (Additional File 3). The sequence conservation index demonstrated a positive performance (AUC 0.74) in agreement with previous reports about structural conservation of conserved lncRNAs [42,43]. However, its performance is lower than the e-value and it is not able to filter out low complexity regions. Therefore, we did not use this measure in the rest of the analyses.

**Table 1:** LncRNAs conservation

Dataset	Word Size	Number conserved lncRNAs	Percentage conserved lncRNA	Percentage conserved shuffled
CNS (239)	11	11	4.60%	0.0%

	10	11	4.60%	0.0%

	9	11	4.60%	0.0%

	8	11	4.60%	0.0%

NCNS (420)	11	23	5.40%	0.0%

Ensembl (2,147)	11	250	11.6%	0.0%

The number of lncRNA putatively conserved in each dataset (CNS, NCNS, Ensembl) after applying the query alignment length and e-value cutoffs on the produced alignments.

### Comparison of the genomic contexts of mouse lncRNA and fish phastCons pairs predicted to be conserved

In order to evaluate the locations and shed light on the putative functions of each conserved fragment, we mapped and compared each element in the respective genic context of both analyzed organisms. The 11 putatively conserved lncRNAs in the CNS dataset showed homology to 10 phastCons elements. The NCNS dataset had 23 lncRNAs showing homology to 21 phastCons and the 250 conserved Ensembl lincRNAs showed homology to 209 fragments from 197 phastCons elements. The conserved regions in zebrafish were checked for overlapping features (Table 2, 3). The fragments from the CNS dataset show 6 out of 10 elements overlapping non-coding regions (intergenic, intronic or non-coding exon) in zebrafish and 4 out of 11 in mouse. Regarding the NCNS dataset, 17 out of 22 conserved sequences are present in a non-coding region in zebrafish and 13 out of 23 in mouse. The situation for the conserved Ensembl dataset is different as a minor fraction of elements is present in non-coding regions in zebrafish and in mouse (18% in zebrafish and 27% in mouse). Such difference with the Ensembl data may be explained by considering that CNS and NCNS lncRNAs are curated for being mainly intergenic as compared to the Ensembl lncRNAs. Therefore, in the Ensembl dataset, candidate lncRNA fragments may overlap an external exon of a coding gene in the same chromosomal domain more frequently. However, they must still be considered non-coding because the orientation of the transcripts is in antisense to the protein coding genes they partially overlap (see genes ENSMUSG00000060029 and ENSMUSG00000046413 as an example) and they do not show any significant open reading frame (ORF). Antisense transcripts are reported to be present in large numbers in mammalian genomes and often linked to the regulation of neighboring or overlapping protein-coding genes [44]. Indeed, long non-coding RNAs can influence the expression of protein coding genes in *cis *as suggested in a previous report [31]. They are also reported to be associated with enhancers of neighboring coding genes in mouse neurons [45] and human [13]. In order to test if the function of flanking coding genes corroborates the functional conservation suggested for each mouse and zebrafish conserved non-coding pair we identified the coding genes flanking and overlapping the aligned region in zebrafish and their mouse counterparts and evaluated their homology relationships. The search for orthologs was performed scanning a window of 1 megabase flanking the conserved elements in either direction in the 2 genomes (see methods). Results are depicted in 3. The figure shows the percentages of conserved mouse lncRNAs sharing orthologous coding gene in the corresponding zebrafish genomic context. All the lncRNA conserved fragments showed at least one ortholog pair from the CNS, the NCNS along with 80% of the Ensembl datasets supporting the hypothesis of syntenic conservation.

**Table 2:** Genomic location of conserved regions in mouse

Dataset	Total aligned regions	Coding exon overlap	Noncoding exon overlap	Intron overlap	Intergenic
CNS	11	7	0	3	1

NCNS	23	10	3	5	5

Ensembl	250	183	31	17	19

The genomic locations for the number of mouse lncRNA fragments found to be conserved. The location is deduced with respect to the coding region of the mouse genome in the area of alignment.

**Table 3:** Genomic location of conserved regions in zebrafish

Dataset	Total aligned regions	Coding exon overlap	Noncoding exon overlap	Intron overlap	Intergenic
CNS	10	4	0	1	5

NCNS	21	4	2	3	12

Ensembl	209	171	6	9	23

The genomic location for the number of phastCons fragments found to be conserved. The location is deduced with respect to the coding region of the zebrafish genome in the area of alignment.

**Figure 3:**
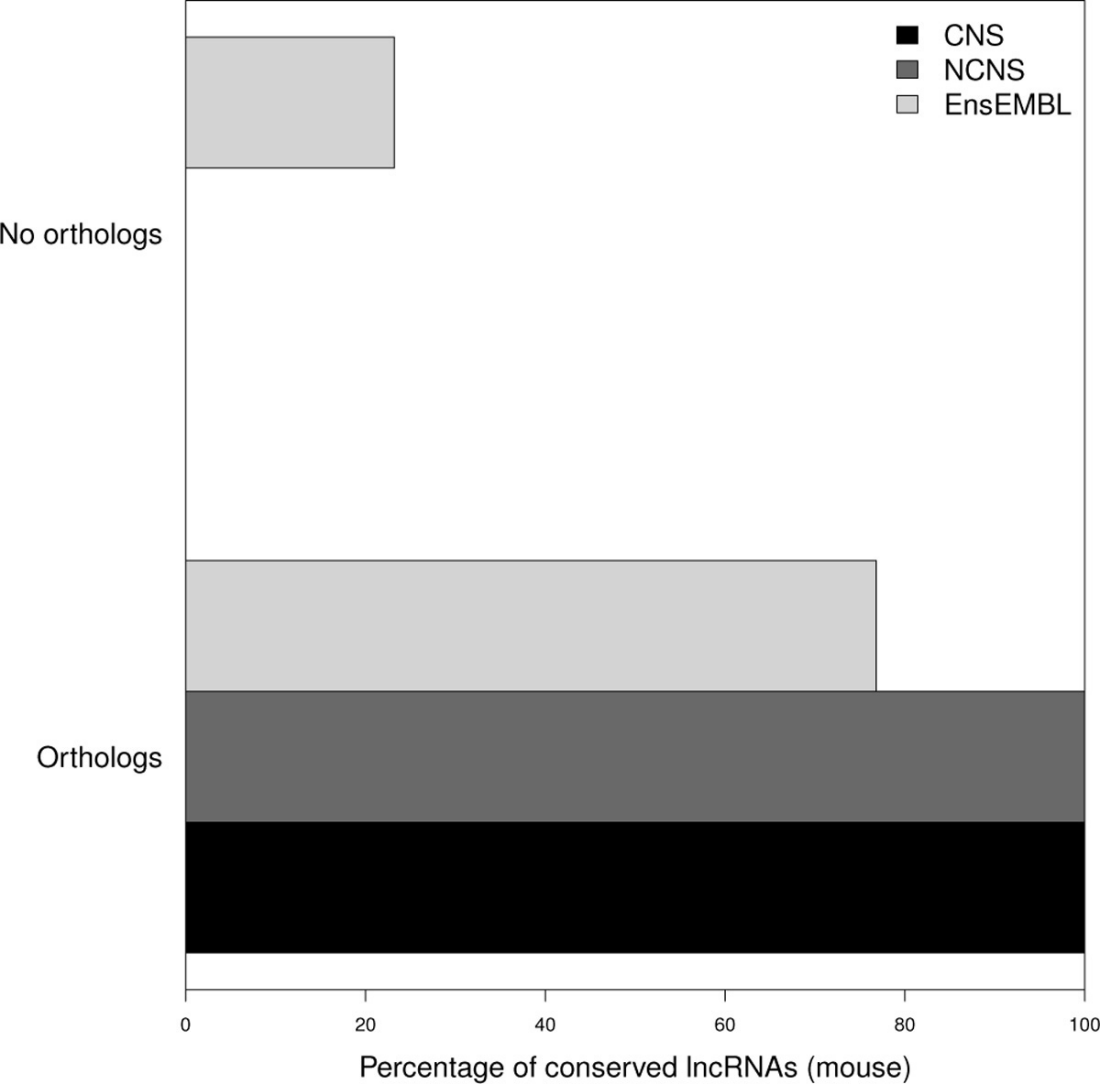
**Orthologous protein coding genes flanking and/or overlapping conserved lncRNAs**. The figure shows the percentage mouse lncRNAs from CNS, NCNS and EnsEMBL datasets, conserved with a zebrafish phastCons element and sharing orthologous coding genes flanking or overlapping the region of conservation in zebrafish.

### Functional enrichment analyses of the protein coding genes proximal to the conserved regions

**Figure 4:**
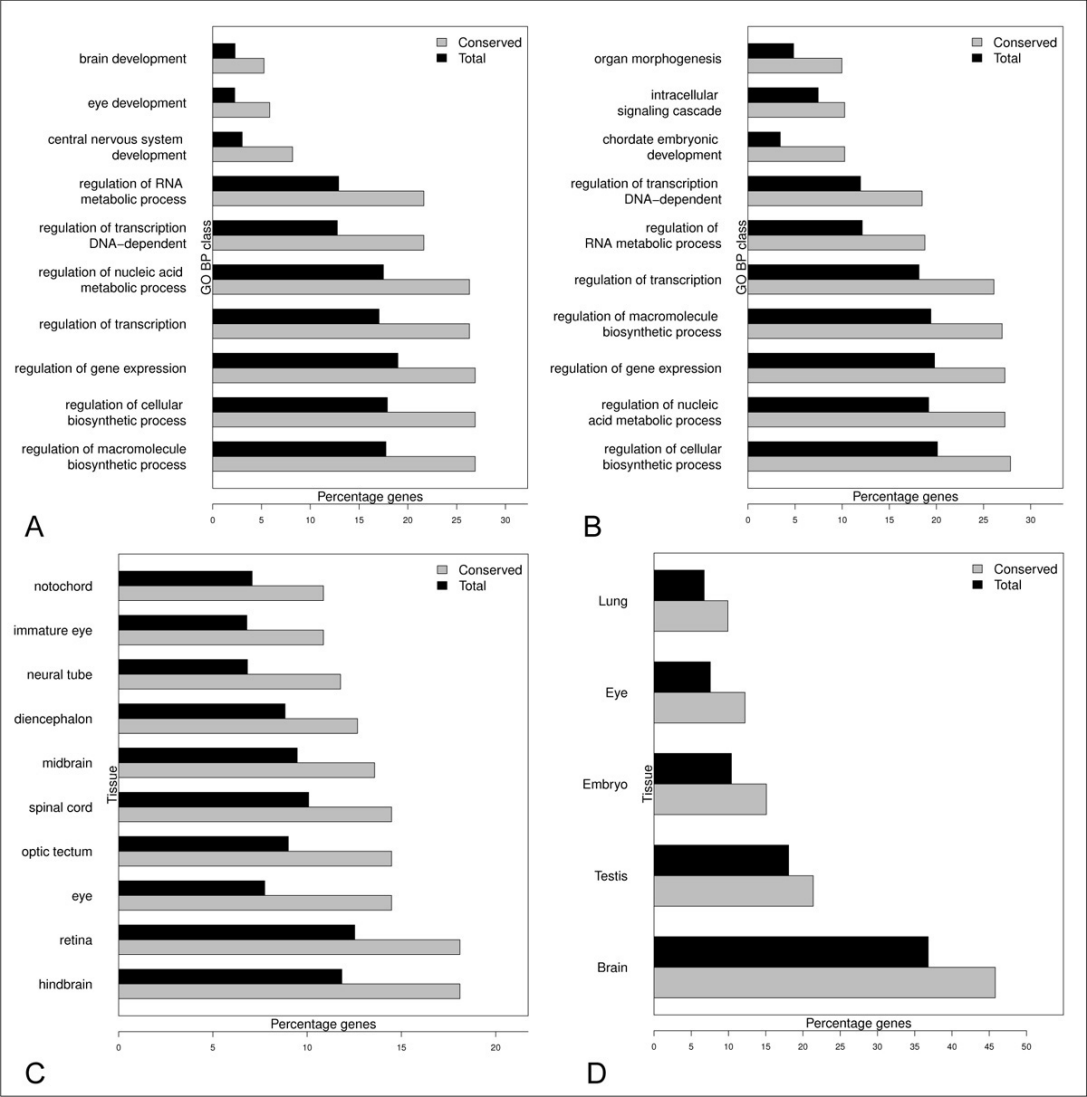
**Function and expression of proteins flanking the conserved elements of the Ensembl dataset**. GO biological process term (level 5) enrichment of **A) **flanking proteins of conserved elements in zebrafish **B) **flanking proteins of conserved elements in mouse for the Ensembl dataset. Tissue enrichment of **C) **flanking proteins of putative conserved elements in zebrafish **D) **flanking proteins of conserved elements in mouse for the Ensembl dataset. **A, B, C, D**: GO terms and tissue of expression are listed only if they are significantly over-represented according to the EASE score. At maximum the 10 top-scoring significant classes are present into the plots. Grey bars indicate the percentages of genes associated to the respective functional classes from the group of genes flanking the identified conserved elements. Black bars indicate the percentages from the entire transcriptome of the given species.

In order to understand the potential biological role of the identified sequences we performed gene ontology and tissue specific expression enrichment analyses on the coding genes flanking the conserved fragments for the Ensembl dataset. The coding genes flanking and eventually overlapping the conserved regions were considered to be the putative lncRNAs associated genes. Significantly enriched GO biological process categories and tissue of expression for the conserved lncRNAs of the Ensembl dataset in zebrafish and mouse were considered for the analysis using DAVID [46,47] and an EASE score cutoff of 0.05. The EASE score is a *p*-value adjustment method specifically designed for biological large-scale studies. It penalizes the significance of categories supported by few genes and favors more robust categories in respect to the Fisher exact probability. It is more conservative than the pure Fisher exact probability and less conservative than the Benjamini and Hochberg FDR [48]. For genes associated to the conserved lncRNAs of the Ensembl dataset, the enriched GO terms included development, regulation of transcription and nucleic acid metabolism in both the organisms (Figure 4A and 4B) in agreement with previous reports in mouse [6,49-51]. Tissue enrichment analyses were also performed to check if the selected genes showed an enrichment for being expressed in similar specific tissues. From this analysis neural and developmental related tissues resulted to be enriched in both the species (Figure 4C and 4D). These results are consistent with previous studies showing that lncRNAs play an important role in regulation, neural development and plasticity [49,50]. It is important to point out that, in mouse, the genes associated to the conserved lncRNAs show the most significant enrichments for expression in the nervous tissues but also a significant enrichment in lung indicating either a possible sub-functionalization of subgroups of lncRNAs or a richer annotation of the mouse transcriptome in terms of domains of expression. Taken together, these analyses highlight a conserved pattern of functions and expression domains of coding genes associated with conserved lncRNA fragments. CNS and NCNS datasets were not used independently because they are not of reasonable dimensions to perform enrichment analysis, however, if we join the 2 datasets together, similar enrichments are obtained (Additional File 4). In 2004 the presence of ultra conserved elements (UCEs) in the human genome was discovered. These elements show about 100% of sequence identity with mouse and many of them are conserved also in fishes. UCEs are greater than 200 nucleotides in length and observed to lie proximal to coding genes related to development, regulation of transcription [52] and cancer related loci [53]. A small fraction of them overlap protein coding exon, however UCEs are mainly non-coding in nature and, although a large fraction seems to be transcribed and/or to function as enhancer they do not overlap current collections of transcripts [53-55]. In order to check if the identified sequences might belong to the ultraconserved family of elements we checked their overlap with UCEs [52,56]. In total four UCEs were found to overlap conserved regions from lncRNAs of the EnsEMBL dataset while a single lncRNA from the NCNS dataset showed overlap with a single UCE. We conclude that the conserved regions identified in this study are not enriched for and do not correspond to UCEs elements. Therefore, they have not to be considered ultraconserved.

### Potential of expression of conserved regions in zebrafish

**Figure 5:**
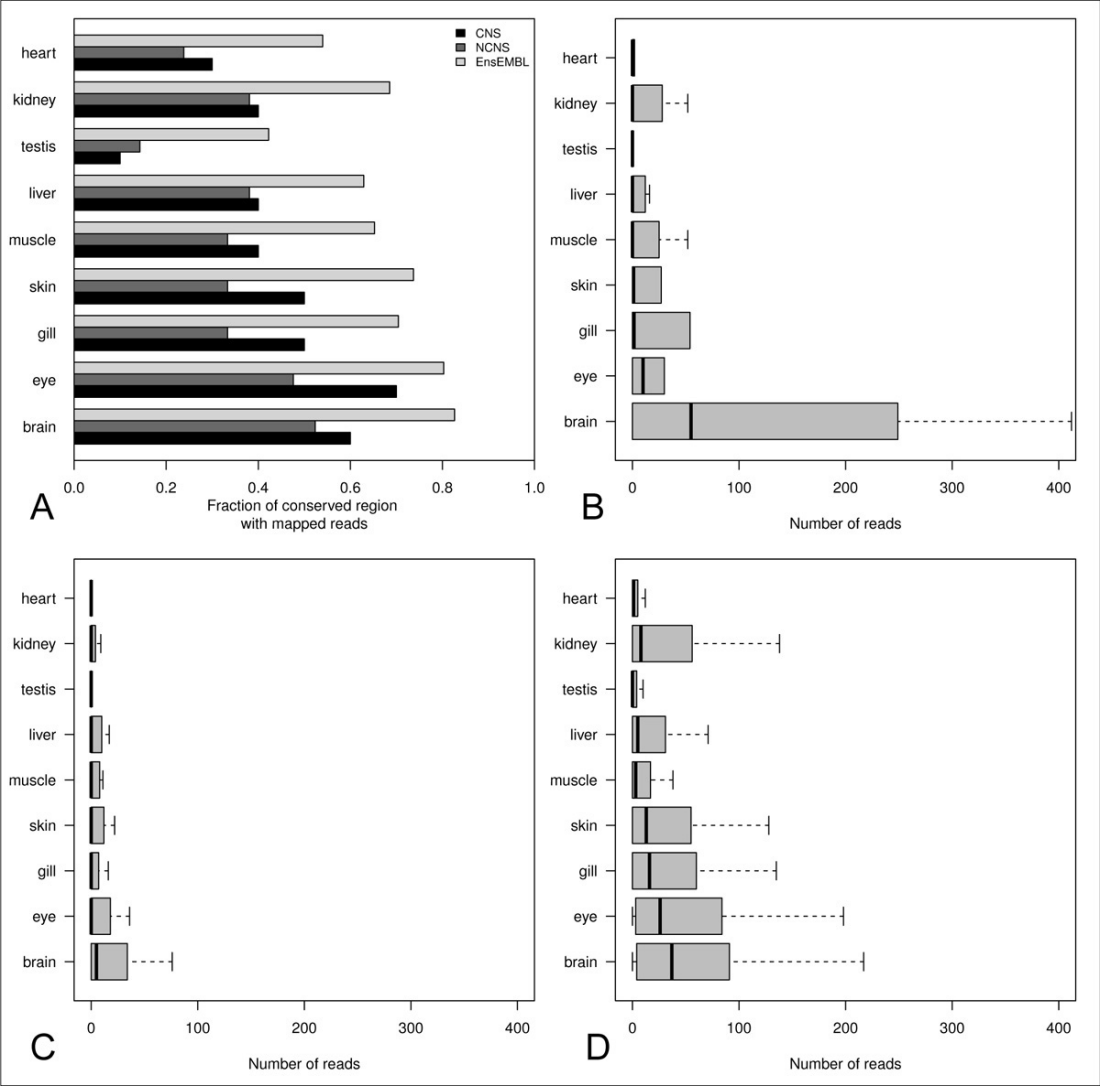
**Tissue specific expression of conserved zebrafish regions mapped on the stickleback genome**. **A) **Fraction of conserved regions in the CNS, NCNS, EnsEMBL datasets showing overlapping RNAseq reads from specific tissues of the stickleback **B) **Boxplot representing the number of reads from each tissue mapping on each conserved region coming from CNS dataset **C) **Boxplot representing the number of reads from each tissue mapping on each conserved region coming from the NCNS dataset **D) **Boxplot representing the number of reads from each tissue mapping on each conserved region coming from EnsEMBL dataset. Boxplots do not show outliers.

The presence of expressed sequence tags (ESTs) overlapping the region of conservation might support an active transcriptional output in the given region. In this context we chose to check for the overlap of zebrafish ESTs in the region of conservation. Respectively 60%, 45% and 70% of the predicted CNS, NCNS and Ensembl conserved regions are covered by at least one EST in zebrafish (Table 4). Interestingly, by randomly selecting ~1,200 non-repeated genomic regions of the same extension from the zebrafish genome we obtained a percentage as small as 8% in overlap with ESTs (*p*-value: 7.5e-08, 5.2e-09 and <2.2e-50 respectively for CNS, NCNS and Ensembl dataset). The results are consistent with the possibility that the majority of the conserved regions predicted in the analysis represent actively transcribed regions of the zebrafish genome. Further, in order to add supporting evidences to the potential of expression of the zebrafish conserved fragments herein isolated we performed overlap analysis with the recently published zebrafish candidate lncRNAs resulting from RNAseq experiments [17,18]. The comparison of all the predicted conserved regions with the published lncRNAs resulted in 6% of our conserved regions showing overlap with at least one reported lncRNAs. It is important to point out that no definitive estimation of the number of lncRNAs expressed in an organism is currently possible. Such uncertainty arises from the fact that non-coding RNAs are expressed at lower levels as compared to coding genes [17,25,57]. Computational identification of lncRNA transcripts from next-generation sequencing data remains a *"work in progress" *in terms of mapping reads to the genome, assembly of new transcripts, definition of background noise and cut-off parameters. Hence, in our analysis a lack of overlap does not signify an absence of transcribed elements in zebrafish, but may reflect on undetected transcripts. In order to test this hypothesis we mapped the raw reads from the study [17] (SRP009426) on the zebrafish genome and computed the overlap between the mapped reads and all the conserved fragments. Interestingly, more than 90% of the predicted conserved regions in the zebrafish genome show overlap with at least one mapped read while only 25% of a set of randomly chosen genomic regions overlap at least one read (*p*-value for difference in proportions <2.2e-50). Checking for regions with more than 1,000 reads overlap, we found that 20% of the conserved regions resulted positive while only 4% of random regions showed such an overlap (p = 1.2e-15; Additional File 5). The highly significant differences between the conserved regions and the random sequences indicate that the RNAseq data supports transcriptional evidences in zebrafish for most of the regions predicted to be conserved lncRNAs. Finally, in order to get information about the expression domains for the conserved sequences we took advantage of the publicly available RNAseq study SRP012923. This study contains RNA samples from nine different tissues (heart, kidney, testis, liver, muscle, skin, gill, eye and brain) of *Gasterosteus aculeatus *(stickleback) a sequenced teleost fish. First, we mapped the conserved zebrafish fragments on the stickleback genome and, as expected, all the zebrafish sequences were mapped. Then, we mapped all the raw reads from the SRP012923 study on the stickleback genome and calculated the overlap with the conserved fragments. Again, more than 85% of conserved elements resulted to overlap raw reads (Figure 5 A) consistently with what we observed in zebrafish using the data from Pauli et al. The stickleback data were generated and made publicly available by the Broad Institute. In agreement with the mouse expression data of the corresponding lncRNAs, the conserved CNS sequences show high levels of expression in the brain (Figure 5 B) also in stickleback. Conversely, the NCNS data result to be transcribed at very low or even background levels (Figure 5 C), while the Ensembl dataset shows low but widespread expression (Figure 5 D). Based on these results we can confirm that the mouse CNS elements are likely to be CNS specific also in fishes. Regarding the NCNS dataset we cannot propose conservation of expression between mouse and stickleback, more and deeper sequencing data will probably clarify this aspect. Finally, the observation that the Ensembl sequences show positive expression levels in several tissues is consistent with the fact that the corresponding mouse transcript models are based on transcriptional evidences from multiple tissues and cell cultures [37]. We conclude that the analyses of reads coming from teleost fishes provides supporting evidences for the transcription and, at least for the CNS specific elements, the tissue specific expression of the predicted conserved regions. As long as new sequencing datasets will become available further light can be shed to improve our knowledge about the similarity of the transcriptional outputs among different vertebrate species.

**Table 4:** ESTs overlapping the region of conservation

Dataset	Conserved	EST overlap
CNS	10	6

NCNS	21	10

Ensembl	209	149

Number of conserved fragments overlapping ESTs in the zebrafish genome.

### CNS specific lncRNA

**Figure 6:**
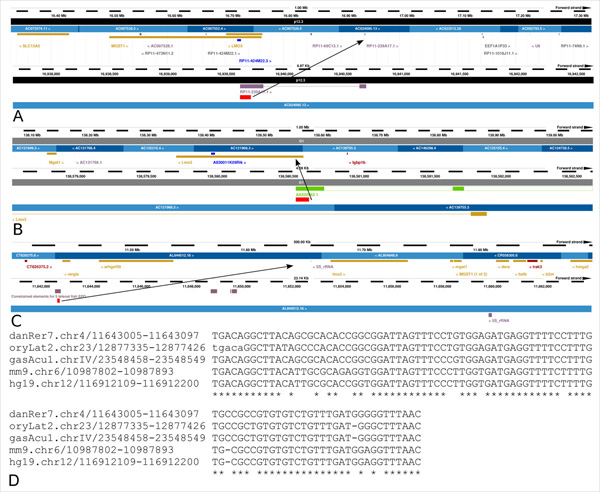
**Genome browser screen-shots for a predicted conserved lncRNA**. **A) **The region conserved (113364) between mouse and zebrafish mapped on the human genome and its position relatively to the *LMO3 *gene. The region of conservation overlaps the exon of a lincRNA predicted by Ensembl in the human genome. **B) **The putative conserved lncRNA in the mouse genome. The red box from which the arrow departs is the conserved fragment 113364. Green boxes above the conserved element mark the exons of the lncRNA AK020962. The conserved element lies inside an intron of an alternative transcript of the *Lmo3 *gene. **C) **Conserved region of the element 113364 in the zebrafish genome indicated by the red box. Grey box above the element represents the region of conservation in teleost fishes. The arrow indicates the position of the element in respect to the *lmo3 *gene in a bigger genomic interval. **D) **Multiple alignment of the region of conservation of the mouse lncRNA with corresponding sequences in human, zebrafish, medaka and stickleback. Note: Arrow marks the region conserved in all species.

To better demonstrate the utility of our analysis we focused on 11 conserved CNS lncRNAs and selected a candidate element ideal for further functional validations. Each phastCons element was given a unique ID at the start of the analysis which is used here as reference (Table 5). The majority of elements show evidence of transcription by overlap with EST sequences in zebrafish and mouse. Many elements also overlap UTRs of protein coding genes pointing towards a putative regulatory function. The conserved element 113364 (Figure 6) belongs to the CNS dataset, it falls completely in an intergenic region in zebrafish and a small part of the lncRNA in mouse overlaps the UTR intron of the coding gene *Lmo3*. The zebrafish sequence shows a conservation of 96 base pairs with the murine lncRNA AK020962 at an e-value of 4e-21 and 88% identity. The l*mo3 *gene flanks the region of conservation in zebrafish too. This gene is known to be a transcriptional regulator [58] and is reported to be involved in cell proliferation and differentiation during embryonic development [59]. It is also implicated in neuroblastoma through its interaction with the neuronal transcription factor *hen2 *[59]. The dataset of conserved elements isolated in our analysis will be of help in focusing on specific sets of elements that might have an evolutionary conserved role in development and differentiation which led to their sequence conservation across species.

**Table 5:** Genomic features of conserved regions overlapping mouse CNS specific lncRNAs

ID	Genomic feature overlap in Zebrafish (Aligned region of phastCons element)					Genomic feature overlap in Mouse (Whole mouse lncRNA)				
	UTR	Exon	Intron	EST	Other	UTR	Exon	Intron	EST	Other

334146	yes	yes	-	yes	-	yes	-	-	yes	lincRNA (Ensembl)

377442	yes	-	-	yes	-	yes	-	-	yes	-

391744	yes	-	-	-	-	yes	-	-	yes	-

414089	yes	-	-	yes	-	-	-	-	yes	-

759212	-	-	yes		-	-	-	yes	yes	-

113364	-	-	-	-	-	yes	-	-	-	-

208793	-	-	-	yes	-	yes	-	-	yes	miRNA

268839	-	-	-	-	-	yes	-	yes	yes	-

460295	-	-	-	yes	-	yes	-	-	yes	-

604458	-	-	-	yes	-	-	-	yes	yes	-

The genomic features for each of the 10 conserved phastCons fragments (from the CNS dataset) and their mouse lncRNA homologs. To be noted that while for zebrafish only the region of alignment is considered not the whole phastCons element, in case of mouse the complete lncRNA was used to perform the analysis. Each region is classified in terms of it being overlapped by a UTR, exon or intron of an annotated protein coding gene, or an EST.

## Conclusions

Long non-coding RNAs are not characterized by the same sequence conservation properties as protein coding genes. However, in our analysis we demonstrated a systematic procedure to identify significant sequence conservation of candidate lncRNAs in vertebrates. It resulted in the selection of a set of mouse lncRNA fragments significantly conserved in fish genomes demonstrating that a proportion of up to 11% of mouse lncRNAs contains fragments conserved across vertebrates. The candidate lncRNAs reflect a sub-population of the mouse lncRNAome sharing sequence homology with zebrafish phastCons elements. The addition of annotation layers on top of sequence conservation statistics provides biological significance to the results obtained. GO enrichment analyses of coding genes flanking the conserved sequences showed striking similarity at the functional level in both species. In addition, significant neural expression enrichments in both mouse and zebrafish are consistent with previous observations that lncRNAs play an important role in neural development, differentiation and functions. The presence of overlapping ESTs and the significant overlap with developmental zebrafish RNAseq reads provide further support that these fragments produce transcriptional output in fishes. Finally, the RNAseq reads from multiple tissues of the stickleback throw light on the tissue specificity of the conserved regions as well as add additional evidences in support of their expression in fishes. The dataset presented constitute a valuable starting point for future studies aimed at functional characterization of specific lncRNAs.

In summary, this work represents a proof of principle showing that a portion between 4 and 11% of lncRNAs retains short regions of conservation between mammals and fishes and the cutoffs to use to efficiently look for such elements. The analyses can result especially useful as a reference to analyze the conservation of lncRNAs in newly sequenced genomes and transcriptomes.

## Methods

### Selection of the datasets used for the study

The mouse CNS (Central Nervous System specific) and NCNS (non Central Nervous System specific) constrained lncRNA datasets were obtained from a previous study [31]. Ensembl lincRNA dataset was obtained from BioMart and is based on the Ensembl version 62 [37,60]. The lncRNA sequences in each dataset were shuffled with the shuffle program (part of the SQUID C library by Sean Eddy, the executable can be found in the HMMER3 program) [61]. Each sequence in each dataset was randomized 100 times giving rise to three random sequence datasets rCNS, rNCNS and rEnsembl. PhastCons elements for zebrafish (zPHS) were obtained from the UCSC table browser [35,62] with the "most conserved" option selected for sequence retrieval. The coordinates of the phastCons elements were mapped to the zebrafish current genome build (zv9) using the UCSC liftover tool [63]. A total of 816,471 conserved elements could be mapped out of 881,975 original elements.

### Identification of sequence homology between the lncRNAs and the phastCons elements

The mouse lncRNAs (CNS, NCNS, Ensembl) as well as the random datasets (rCNS, rNCNS, rENsEMBL) were searched individually against the zPHS using BLASTn from the BLAST+ software package (version 2.25) [64]. All the BLASTn parameters were kept default except for the word size. Parsing of the blast results was carried out in a pipeline using custom perl scripts. BLASTn searches for word sizes from 8 to 11 were executed for the CNS specific lncRNA and rCNS datasets against the phastCons elements. The NCNS/rNCNS and Ensembl/rEnsembl datasets were queried against zPHS at word size 11. Four parameters from the BLASTn search results were considered in the ROC analyses: query coverage (fraction of a lncRNA which is aligned to a phastCons element), alignment length (the length of the alignment including the gaps inserted), percentage identity (number of identical base matches between the query and the subject sequences) and e-value (a score which defines the probability of an alignment not being random in nature). The alignments of the lncRNAs (CNS/NCNS/Ensembl) against the zPHS were taken as the true positive dataset while those from the randomized datasets (rCNS/rNCNS/rEnsembl) were considered to be the false positive set. The ROCR package in R environment was utilized to build the receiver operating characteristic (ROC) curve of false positive against true positive values for each parameter [65]. ROC curves for the e-value parameter in the plots show the reciprocal of the e-value (1/e-value) because plotting the e-value produced curves sensibly skewed below the diagonal line. Each alignment produced from the BLASTn search of the CNS dataset against zebrafish was also considered for structural conservation analysis. SISSIz program [66] was used to randomize each alignment 100 times using a dinucleotide model (SISSIz --simulate --tstv -n 100) to generate a randomized alignment dataset to measure the structural conservation (srCNS). The alignments of the CNS and srCNS datasets were checked for structural conservation with the RNAz 2.0 software (default parameters) [41]. To build ROC curves we used the following parameters from the RNAz output: ratio of pairwise identity by sequence conservation index, Z score and P values (1/P value). The parameters from the original alignments were considered to be true positive while those from the randomized alignments were considered to be the false positive. ROC curves of the false positive against the true positive were plotted for each the parameter.

### Genomic features identification and enrichment analysis

The predicted conserved mouse lncRNAs were obtained after using the e-value and query alignment length thresholds as defined by the ROC curves in order to have less than 0.05% false positives passing it. The conserved lncRNAs (named cCNS, cNCNS, cEnsembl) and their respective zPHS elements sharing sequence similarity (named zCNS, zNCNS, zEnsembl) were back mapped to the mouse and zebrafish genomes (mm9 and zv9) respectively using BLASTn with default parameters but -culling_limit = 1. The mapped coordinates of each mouse lncRNA and zebrafish conserved element were used to retrieve overlapping genes, transcripts, exons, and the closest flanking protein coding genes in a window up to 1 mb using custom perl scripts which use the Ensembl core modules API [67] and programmatic access to the Ensembl databases version 62. DAVID gene annotation tool was used for the GO term enrichment and tissue expression enrichment analyses for the protein-coding genes flanking and overlapping the conserved elements using the whole transcriptome as universe [46]. An EASE score of 0.05 [48] was used as a cut-off for the enrichment analysis. Sequences of ultraconserved elements [52,56] were mapped on the mouse genome using BLASTn (-task blastn -culling_limit 1) with default parameters. The coordinates of the mapped elements on the mouse genome were checked for overlap with conserved mouse lncRNAs using overlapBed program from the BEDTools package [68] (version 2.14.2) with default parameters. In all the overlap analyses performed we have considered sufficient an overlap of at least 1 bp between the conserved element and the specific feature considered.

### Identification of orthologs between mouse and zebrafish and mapping of ESTs in the region of conservation

Zebrafish and mouse homology information were downloaded from BioMart [60] based on EnsEMBL version 62. We collected all the Ensembl genes mapped in intervals up to 2 Mb (1 Mb up and down-stream) around each conserved element in both the genomes. For each element we looked for genes considered evolutionary related (classified as ortholog one to one, ortholog one to many or ortholog many to many) in Ensembl Compara [69]. Conserved elements were considered syntenic if showing at least one evolutionary related gene in the given interval for the species considered. The analysis was performed individually on all lncRNAs stemming from the cCNS, cNCNS and cEnsembl datasets. The EST coordinates for mouse and zebrafish were downloaded from UCSC databases on 14^th ^September 2011 [70,71]. The region of sequence conservation in the mouse lncRNAs (cCNS/cNCNS/cEnsEMBL) were checked for the overlap with a reported EST on the mouse genome. The same process was repeated on the zPHS conserved fragments (zCNS/zNCNS/zEnsEMBL) with respect to zebrafish ESTs. The EnsEMBL genome browser was used to generate the images for the conserved zPHS region 113364 [72] and its corresponding lncRNA in mouse as well as the region in human showing sequence homology to 113364.

### Mapping of RNAseq data and read count on conserved regions

The zebrafish paired end RNAseq data from 7 developmental stages and stickleback paired end RNAseq from 9 tissues were downloaded from the European Nucleotide Archive in the fastq format (Accessions: SRP012923 and SRP009426). The raw reads were mapped to the zebrafish and stickleback genome using Tophat 2.0.4 [73] (tophat -p -o -G) and overlap associations for the conserved regions were calculated using custom perl scripts and the coverageBed (coverageBed -split -aBam -b) program from the BEDTools package [68] (version 2.14.2). Zebrafish sequences of the conserved elements were mapped on the stickleback genome using BLASTn (-task blastn -culling_limit 1) with default parameters and all the regions were mapped with a minimum percentage identity of 70%. Random regions (~1,200) on the zebrafish genome were selected using the shuffleBed (shuffleBed -i -g) program from the BEDTools package. Overlap associations for the random regions were calculated in the same way as that for conserved regions.

## List of abbreviations

lncRNA: long noncoding RNA; EST: expressed sequence tag; UCE: ultraconserved element; GO: gene ontology; CNS: central nervous system specific; NCNS non-central nervous system specific; ROC: receiver operating characteristic; AUC: area under curve; FDR: false discovery rate.

## Competing interests

The authors declare that they have no competing interests.

## Authors' contributions

RS and SB conceived the study. SB designed and executed the analysis, interpreted the results and drafted the manuscript. RS supervised the study, participated in the design and execution of the analysis, the interpretation of results and helped to draft the manuscript. FM participated in the design of the analysis and interpretation of results and helped to draft the manuscript. All authors read and approved the final manuscript.

## Declarations

The publication costs for this article were funded by the corresponding author's institution.

## Supplementary Material

Additional File 1 **ROC curves of CNS dataset at word size 8-10**. ROC curves for query coverage (QCoverage), percentage identity (PIdentity), query alignment length (QAlength) and e-value (EValue) for the CNS dataset at word size **A) **8 **B) **9, **C) **10. The cut-off for a parameter is defined as the point of steep incline in the true positive rate as compared to the false positive rate. The significant cut-off defined in the present analysis are indicated by arrows. Click here for file

Additional File 2 **Conserved lncRNAs**. Results of BLASTn search and annotations concerning the genomic locations and proximal protein-coding genes of predicted conserved elements in mouse and zebrafish. The excel file contains three data sheets. The data is from CNS, NCNS and Ensembl datasets. The data includes BLASTn search results of the mouse lncRNAs against zebrafish phastCons elements. For each predicted conserved element in zebrafish and mouse information pertaining to its genomic location and overlapping/flanking gene features is provided as obtained from the Ensembl databases. If an element overlaps more than one feature each overlap is reported in a separate row. Click here for file

Additional File 3 **ROC curve for structural conservation of CNS lncRNAs dataset**. **A) **Pairwise identity/Sequence conservation index (AUC 0.74), **B) **Z score (AUC 0.47) and **C) **inverse *P*-value (AUC 0.57) for the **mouse CNS constrained lncRNAs **against the zebrafish phastcons elements. Click here for file

Additional File 4 **Function and expression of proteins flanking the conserved elements of the CNS and NCNS dataset**. GO biological process term (level 5) enrichment of **A) **flanking proteins of conserved elements in zebrafish **B) **flanking proteins of conserved elements in mouse for the CNS and NCNS dataset. Tissue enrichment of **C) **flanking proteins of putative conserved elements in zebrafish **D) **flanking proteins of conserved elements in mouse for the CNS and NCNS dataset. **A, B, C, D**: GO terms and tissue of expression are listed only if they are significantly over-represented according to the EASE score. Grey bars indicate the percentages of genes associated to the respective functional classes from the group of genes flanking the identified conserved elements. Black bars indicate the percentages from the entire transcriptome of the given species. Click here for file

Additional File 5 **RNAseq data overlap on conserved zebrafish elements**. The figure depicts the percentage of conserved elements in the zebrafish genome which show overlap with > 1, > 25 and > 1000 short reads (coming from RNAseq of zebrafish development stages) as compared against a set of random elements in the fish genome. Click here for file
