# Supplementary material for: Examples of sequence conservation analyses capture a subset of mouse long non-coding RNAs sharing homology with fish conserved genomic elements
Source: BMC Bioinformatics. 2013 Apr 22;14(Suppl 7):S14. doi: 10.1186/1471-2105-14-S7-S14 (PMC3633045; doi:10.1186/1471-2105-14-S7-S14)
Supplement: Additional File 1 — ROC curves of CNS dataset at word size 8-10. ROC curves for query coverage (QCoverage), percentage identity (PIdentity), query alignment length (QAlength) and e-value (EValue) for the CNS dataset at word size A) 8 B) 9, C) 10. The cut-off for a parameter is defined as the point of steep incline in the true positive rate as compared to the false positive rate. The significant cut-off defined in the present analysis are indicated by arrows. [file 1471-2105-14-S7-S14-S1.pdf]

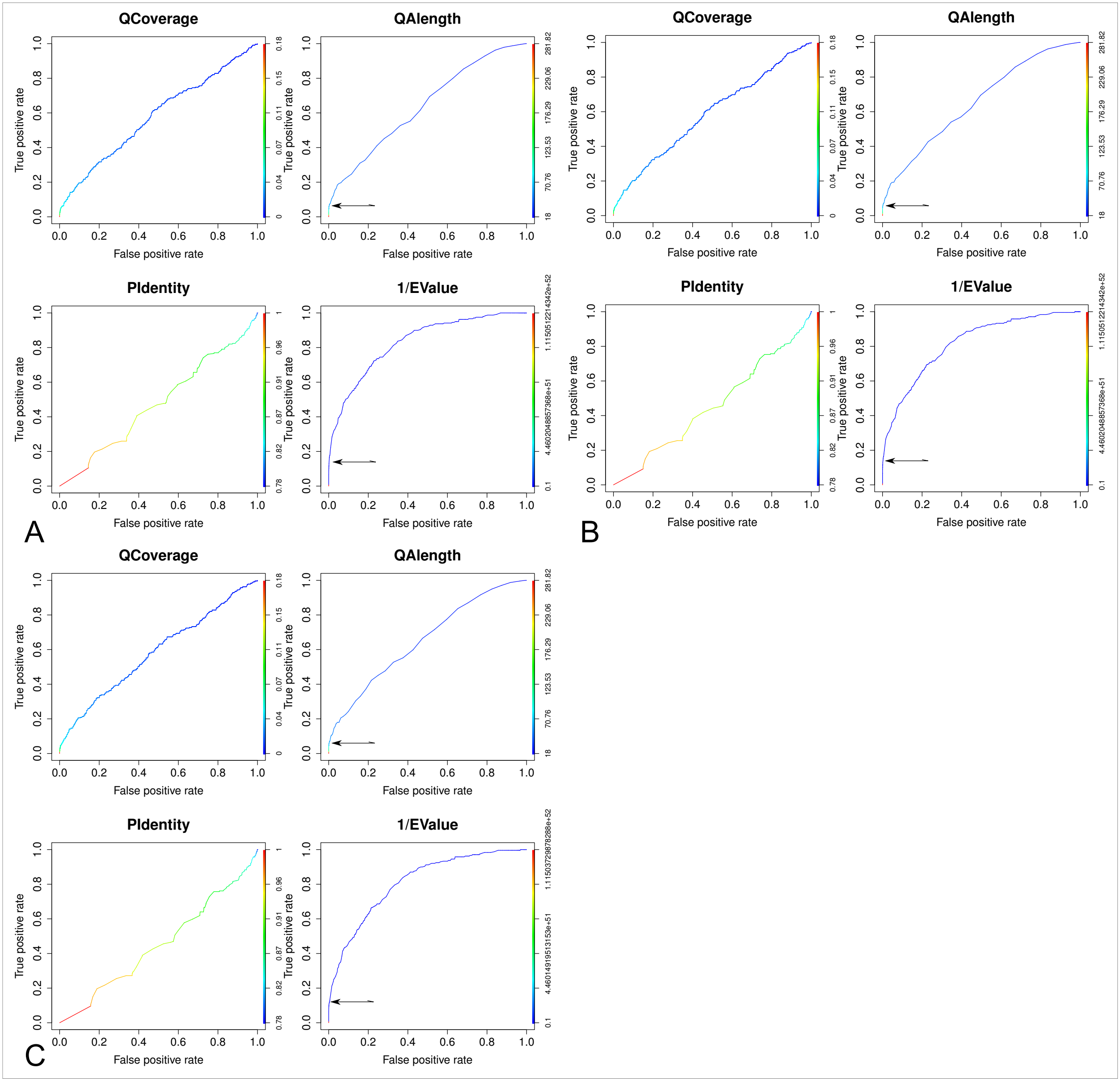
A
QCoverage
True positive rate
False positive rate
0.0 0.2 0.4 0.6 0.8 1.0
0 0.04 0.07 0.11 0.15 0.18
QAlength
18 70.76 123.53 176.29 229.06 281.82
PIdentity
0.78 0.82 0.87 0.91 0.96 1
1/EValue
0.1 4.46020488573680e+51 1.1150512214342e+52
B
QCoverage
QAlength
PIdentity
1/EValue
C
QCoverage
QAlength
PIdentity
1/EValue
0.1 4.46014919513153e+51 1.11503729878288e+52
