# Supplementary figures and images for: Examples of sequence conservation analyses capture a subset of mouse long non-coding RNAs sharing homology with fish conserved genomic elements
Source: BMC Bioinformatics. 2013 Apr 22;14(Suppl 7):S14. doi: 10.1186/1471-2105-14-S7-S14 (PMC3633045; doi:10.1186/1471-2105-14-S7-S14)

**PID/SCI**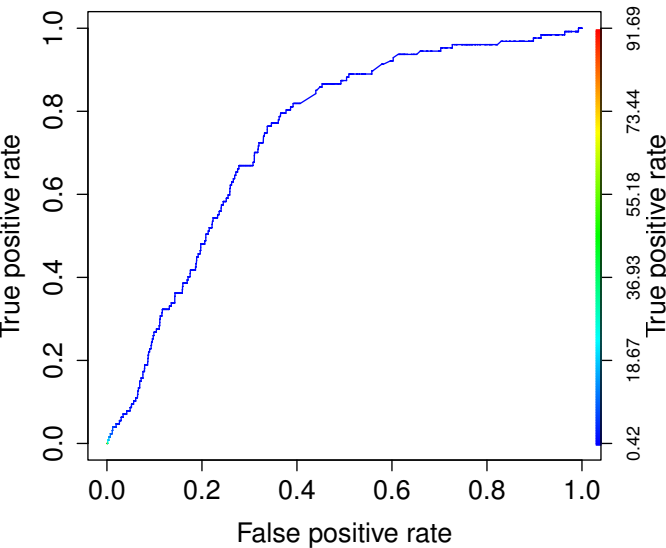**Z.Score**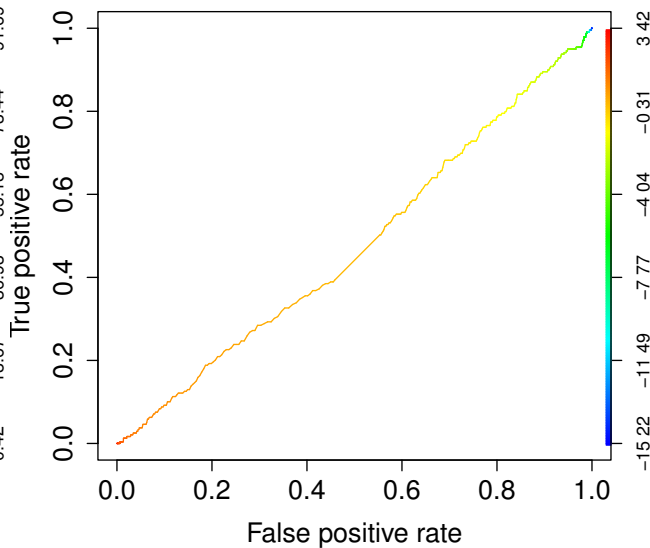**1/P.Val**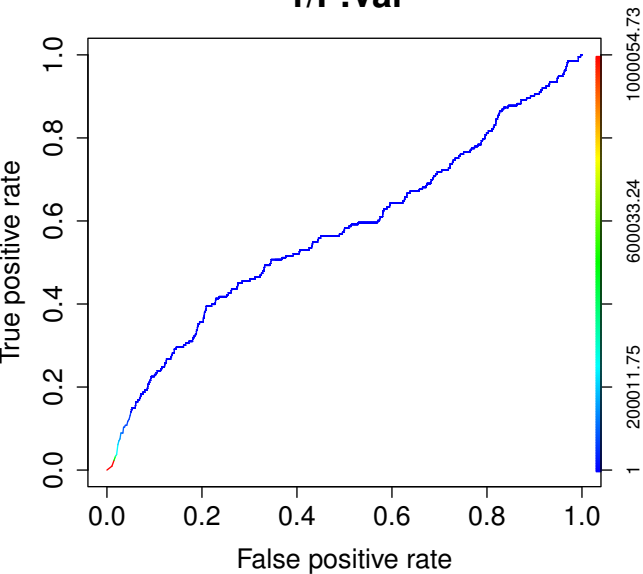

Supplement: Additional File 3 — ROC curve for structural conservation of CNS lncRNAs dataset. A) Pairwise identity/Sequence conservation index (AUC 0.74), B) Z score (AUC 0.47) and C) inverse P-value (AUC 0.57) for the mouse CNS constrained lncRNAs against the zebrafish phastcons elements. [file 1471-2105-14-S7-S14-S3.pdf]

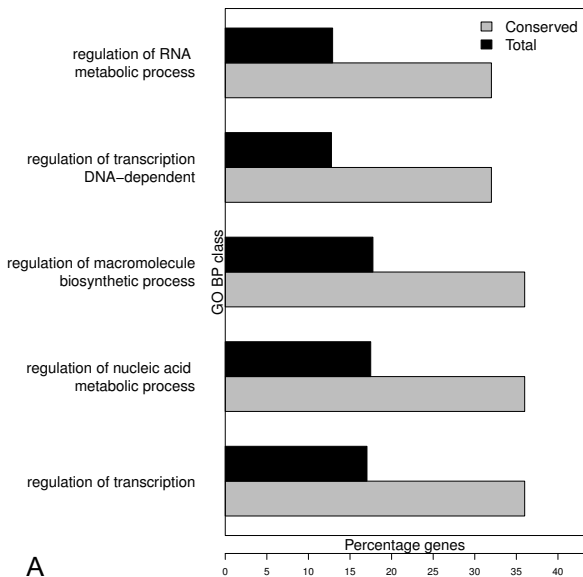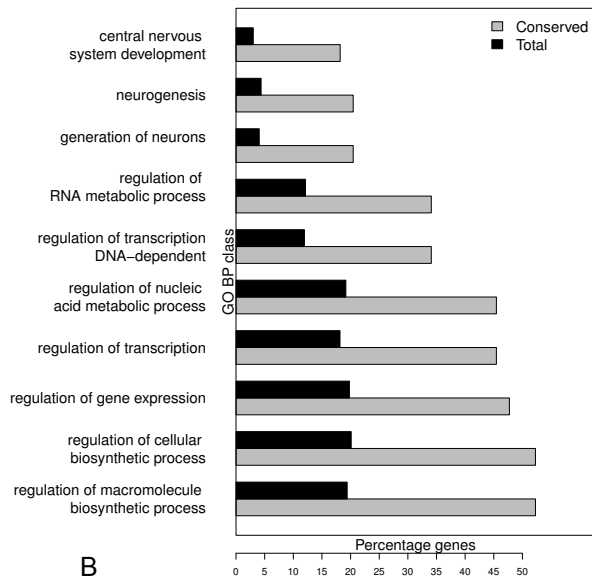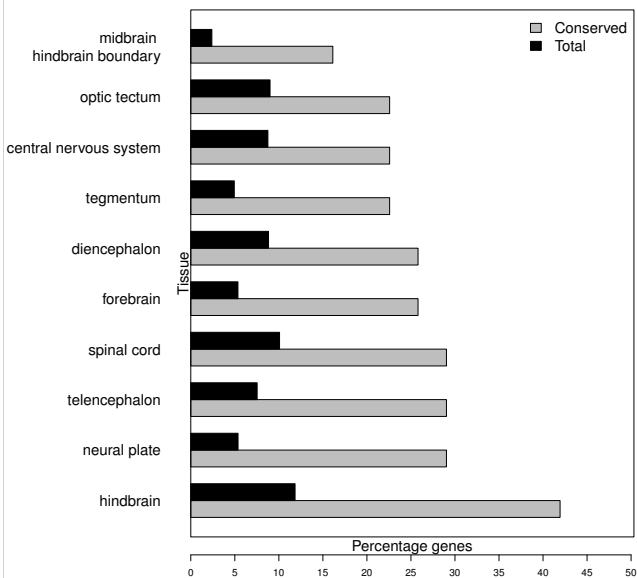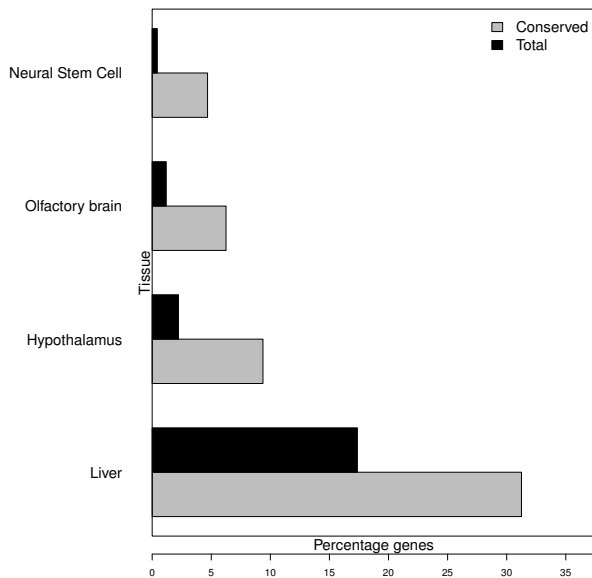

Supplement: Additional File 4 — Function and expression of proteins flanking the conserved elements of the CNS and NCNS dataset. GO biological process term (level 5) enrichment of A) flanking proteins of conserved elements in zebrafish B) flanking proteins of conserved elements in mouse for the CNS and NCNS dataset. Tissue enrichment of C) flanking proteins of putative conserved elements in zebrafish D) flanking proteins of conserved elements in mouse for the CNS and NCNS dataset. A, B, C, D: GO terms and tissue of expression are listed only if they are significantly over-represented according to the EASE score. Grey bars indicate the percentages of genes associated to the respective functional classes from the group of genes flanking the identified conserved elements. Black bars indicate the percentages from the entire transcriptome of the given species. [file 1471-2105-14-S7-S14-S4.pdf]

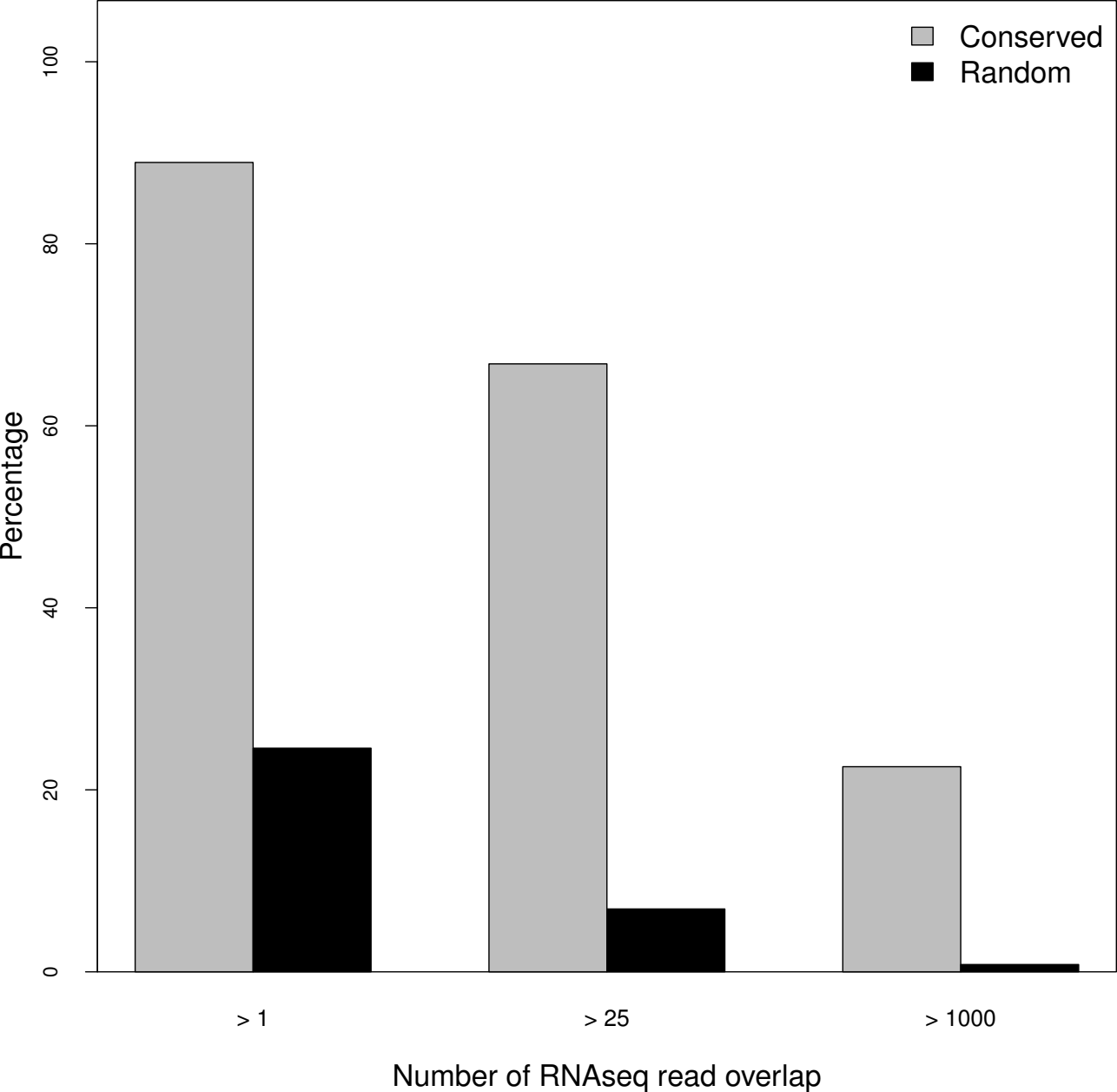

Supplement: Additional File 5 — RNAseq data overlap on conserved zebrafish elements. The figure depicts the percentage of conserved elements in the zebrafish genome which show overlap with > 1, > 25 and > 1000 short reads (coming from RNAseq of zebrafish development stages) as compared against a set of random elements in the fish genome. [file 1471-2105-14-S7-S14-S5.pdf]
